# The impact of the program structure at Hannover Medical School on academic success in medical studies

**DOI:** 10.3205/zma001169

**Published:** 2018-05-15

**Authors:** Volkhard Fischer, Agnieszka Dudzinska, Ingo Just

**Affiliations:** 1 Hannover Medical School, Dean of studies office, Academic controlling, OE 9135, Hannover, Germany; 2 Leibniz-Universität Hannover, Zentrale Einrichtung für Qualitätsentwicklung in Studium und Lehre, Studierenden- & Absolventenbefragungen, Hannover, Germany; 3 Hannover Medical School, Dean of Study, Hannover, Germany

**Keywords:** Student selection, admission groups, program structure, study success, medicine

## Abstract

**Aim:** The classical course structure for medicine in Germany is separated by three sections of the medical state examination. This structure is generally regarded as sensible and unchangeable. Because the special program structure at Hannover Medical School (MHH) has one integrated, rather than two separate study blocks, it is possible to examine the influence of structural modifications on the study success of different admission groups.

**Methods: **The data was obtained from students admitted to the MHH between 2006 and 2008 in different admission quotas. Study success was defined as the successful completion of the entire program, but completion of the first section of the state examination was also analysed.

**Results: **More students from the best “Abitur” (school leaving examinations) quota successfully completed their studies than those accepted via the selection process of the universities. The latter were more successful than students from the waiting list quota. However the successful graduates of this last group completed their studies more often within the prescribed period of study, although they needed more time for completing all parts of the first section of the state examination.

**Conclusion:** The data shows that an integrated course structure can offer, in particular, students from the waiting list quota, the opportunity to compensate for delays in the first years of study. However, they do not provide any evidence which applicants are best suited to meet the social and professional requirements of trained doctors. Due to the complex structure of such longitudinal studies, our results allow more than one plausible interpretation.

## Zusammenfassung

**Zielsetzung:** Der klassische, durch Teilprüfungen des medizinischen Staatsexamens unterteilte Aufbau des Medizinstudiums wird in der Regel als sinnvoll und deshalb unveränderbar hingenommen. Der Modellstudiengang der Medizinischen Hochschule Hannover (MHH) weist statt zwei Studienabschnitten nur einen integrierten fünfjährigen Studienabschnitt vor dem Praktischen Jahr (PJ) auf. In ihm kann deshalb der Einfluss struktureller Modifikationen auf den Studienerfolg verschiedener Zulassungsgruppen beispielhaft untersucht werden.

**Methodik:** Betrachtet wurde der Studienerfolg der über die Vorabquoten und die drei Hauptquoten zwischen 2006 und 2008 an der MHH zugelassenen Studierenden. Studienerfolg wurde dabei als erfolgreicher Abschluss des gesamten Studiums definiert, ohne den Abschluss des ersten Abschnitts der ärztlichen Prüfung (M1) auszublenden.

**Ergebnisse: **Die Studierenden aus der Abiturbesten-Quote schlossen ihr Studium an der MHH im Untersuchungszeitraum häufiger erfolgreich ab als die Studierenden, die über das Auswahlverfahren der Hochschulen an die MHH gekommen waren. Diese waren wiederum häufiger erfolgreich als MHH-Studierende aus der Wartezeitquote. Gleichzeitig waren letztere häufiger innerhalb der Regelstudienzeit mit dem Studium fertig, obwohl sie seltener innerhalb der Regelstudienzeit alle Teile der M1-Prüfung bestanden hatten.

**Schlussfolgerung: **Die Daten verdeutlichen, dass ein integrierter Studienabschnitt vor dem PJ insbesondere für Studierende aus der Wartezeitquote die Chance bieten kann, Studienzeitverzögerungen aus den ersten Studienjahren im weiteren Verlauf des Studiums zu kompensieren. Ein einstufiger Studienabschnitt kann also die Möglichkeit erhöhen, zügiger zu studieren. Auch wenn die Komplexität der Daten verschiedene Erklärungsansätze erlaubt.

## 1. Introduction

### 1.1. National and international context 

Studies on the influence of the type of university admission on how successful medical students are, such as the current one by Heidmann, Schwibbe, Kadmon and Hampe [1], face two challenges: first, they have to reduce a complex system of input, structural and output variables to a few selected comparisons in order to keep research hypotheses interpretable. Secondly, this kind of work requires tenacity, because in medical studies are at least six years and three months between acceptance to university and the earliest possible graduation.

Habitually, medical students accepted to Hannover Medical School (MHH) in four different ways: “Abiturbeste” (students with the highest grades in high school examinations “Abitur”: university entrance qualification), “Wartezeit” (waiting list of approximately 7 years without the possibility of studying other subject), “Auswahlverfahren der Hochschulen (AdH)” (selection process of the universities) and “Vorabquoten” (priority quota; providing study places for special groups of applicants). Using the example of three year groups in the “Modellstudiengang HannibaL” (model study course “HannibaL”) at MHH, it will be demonstrated that not only the admission process, but also the course structure can have a significant influence on successful graduation. The reason that this topic has been neglected might be the time required for modifying basic course structures and their implementations, which can take at least a decade.

In former analyses on the influences of different selection procedures on successful graduation in medical studies, the structure of those medical courses were completely ignored as all faculties trained within the same course structure prescribed by the “Approbationsordnung” (German Medical Licensure Act) [2]. Also, the focus on the M1 examination (formerly known as “Physikum”) appeared obvious, since it was the first central examination, which was taken in every Institution and success in this examination was a good predictor for success in the second stage of the state examination [3]. Nevertheless, those former studies did not reduce “success” to passing the M1 examination (first part of the study course). In international studies, being successful refers to the final degree grade (cf. e.g. [4]), however, at the same time it is emphasized that mere grades should not be the only measured criteria [5].

#### 1.2. Initial situation at MHH

Since the 1990s, student selection and the variety of medical course structures in Germany have become more diverse. The “Hochschulzulassungsrecht” (university admission regulations) for all study courses has been changed to allow more space for other candidate criteria than grade performance alone (cf. for example http://www.schure.de/22220/nhzg.htm). The “Modellklausel” (model clause) (§ 41) of the “Approbationsordnung” of 2002, provided the opportunity to test alternative study structures. In the winter semester 2005/06 the model study course “HannibaL” was introduced and is characterized as follows:

- The preclinical and clinical study sections have been merged into one integrated study section. In each academic year, one to two interdisciplinary modules ensure the integration of preclinical, clinical-practical and clinical-theoretical contents. Already in the first two years of the study course education takes place with patients. In order to switch between the model study course and standard courses, the M1 examination equivalency can be received after two years. The majority of the modules are however aligned with the performance records required by the “Approbationsordnung”.
- In the integrated section of the study course, MHH focuses on the development of a consistent basic framework of knowledge, abilities and skills in all students. This includes, from the first semester on, patient contact on ward rounds for clinical teaching purposes.
- The first part of the medical examination (M1) does not consist of a major written and oral-practical examination, but cumulatively through passing all modules of the first two years of the study course. Hence, every single examination must be passed separately. If a single examination is ultimately failed, this means, the whole M1 has been failed. Table 1 (Tab. 1) shows each separate examination and it´s positioning in the first two years of the study course. By integrating the two sections of the course, students can – with minor limitations – progress in their studies, even if they have not yet completed all parts of their M1 equivalence. This means that in order to participate in modules of study year three to five, one does not need to have passed all modules of the first two academic years. All faculties studied by Heidmann et al. [1], however, require the passing M1 examination (first part of the study course) before clinical classes can be started from the third year onwards.

The 2004 amendment to the “Hochschulrahmengesetz” (Framework law for universities and colleges) and the specification for Lower Saxony in the “Niedersächsische Hochschulzulassungsgesetz” (NHZG) (University admission law for Lower Saxony) in 2005 (current version cf. http://www.schure.de/22220/nhzg.htm), obliged universities to change their “AdH” (selection process of the universities) procedures. In addition to the average grade of the “Abitur” – universities have to take into consideration at least one other criteria which reflects the suitability of the candidates. The MHH decided on selection interviews. In addition to personal qualifications, technical aspects and/or extracurricular interests, applicants have the chance to show their particular motivation for studying medicine.

Compared with other federal states, the MHH has taken almost as much advantage of the narrow room for manoeuvre within the NHZG as is possible. In the framework of the “AdH” process, three times as many applicants as places available are pre-selected on the basis of the average grade of their “Abitur”. These are invited for selection interviews. Taking the total number of points from the selection interview and the scores for the final “Abitur” grade, the weighted average is calculated and serves as a basis for ranking of places (current version cf. [6]). Regarding the other admission quotas (“Vorabquoten”, “Abiturbeste” and “Wartezeit”), universities accept candidates in accordance with common nationwide criteria.

Many, however not all faculties, focus in the “AdH” process on performance parameters. This does not apply to the MHH and its model study course “HannibaL”. Therefore, data concerning MHH students may allow for a critical view of the implicit assumptions made in recent German publications [7], [8].

## 2. Method

Since the NHZG came into force late, there were no selection interviews conducted in the “AdH” process the first year of the model study course. With years three and four, completion of studies within the prescribed period meant they were completed before the start of the survey. Even a delayed graduation could only be illustrated in a limited way. For a more precise analysis, those 307 students from year two were analyzed, since they were the first students admitted to the university via selection interviews and their earliest possible graduation was estimated for Autumn 2012. This also allowed for students who took the second part of the medical examination in autumn 2015, up to three years after the earliest examinations, to also be analysed.

Another reason for looking at the second cohort in detail was, that due to the lack of experience with the “AdH” process in 2006, there was a significant overbooking of spaces and, for this reason, for the first five years of the model study course no lateral entrants were accepted to fill spaces. 

Although the number of career changers at MHH count for less than five per cent of entrants per year, their late entry into the integrated study program naturally has an impact on the various admission groups.

For all students the following variables were evaluated:

- the kind of university entrance (“Vorabquoten”, “Abiturbeste”, “AdH”, “Wartezeit”),
- the “Abitur” average grade,
- time taken to attain M1 equivalence in semesters,
- time needed to successfully pass the M2 examination in semesters, if it was sat by the “Landesprüfungsamt Niedersachsen” (State examinations board of Lower Saxony),
- and the status of enrollment in November 2015.

Additionally, for students admitted to the MHH via the “AdH”-process, it was recorded, whether they were admitted on the basis of the “Abitur” average grades or whether they also needed the points from the selection interview.

This survey should explore whether structural changes in the medical course can have a significant impact on successful graduation. Due to its limitations (only one university, only a few evaluable cohorts), significant differences in the number of people from the different admission groups, a greater number of additional analyses would be required in order to determine the strength of various effects. However, that would move the central question out of sight: Are structural changes to the study course of medicine possible, which lead to more equal chances of successful graduation for students from different admission groups?

## 3. Results

**Figure 1:**
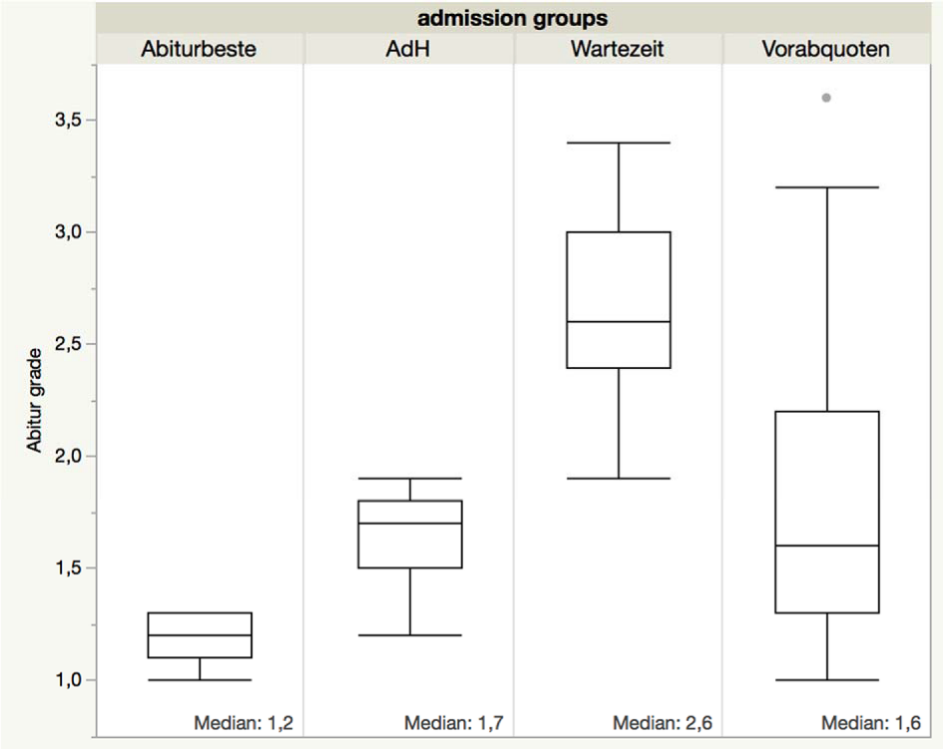
Distribution of ‘Abitur’ average grades for students of the study course years 2006/07 by admission groups.

The “Abitur” average grades were distributed as expected among the different admission groups. The “Abiturbeste” achieved better grades than those entering via the “AdH” process, while the “Wartezeit” students had the lowest grades. The grades of “Vorabquoten” students were distributed across the whole range of admission groups (see Figure 1 (Fig. 1)), since this quota was very heterogeneous (non-EU citizens, students from Federal armed forces and personal hardship cases).

**Figure 2:**
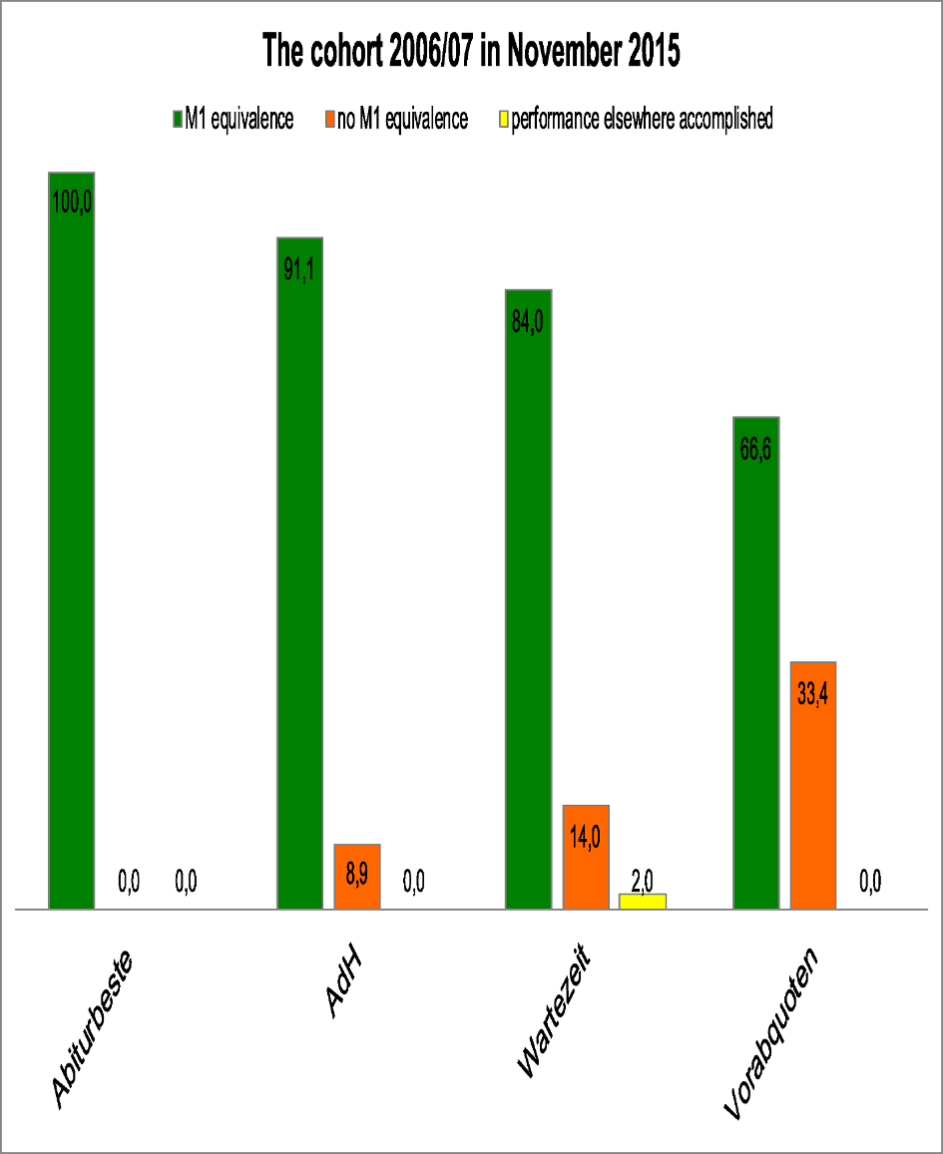
Percentage of students with successful M1 equivalence of the study course year 2006/07 by admission groups (status at Nov 2015).

Nine years after attending the study course, all of the “Abiturbeste” students had received their M1 equivalence. 84% of the “Wartezeit” students had also passed their intermediate examination successfully. As with the “Abitur” grades, “AdH” students achieved results between those of the “Abiturbeste” and “Wartezeit” groups for the intermediate examination. Only the number of “Vorabquoten” students successfully passing the M1 equivalence did not correspond to the “Abitur” average grades (see Figure 2 (Fig. 2)).

**Figure 3:**
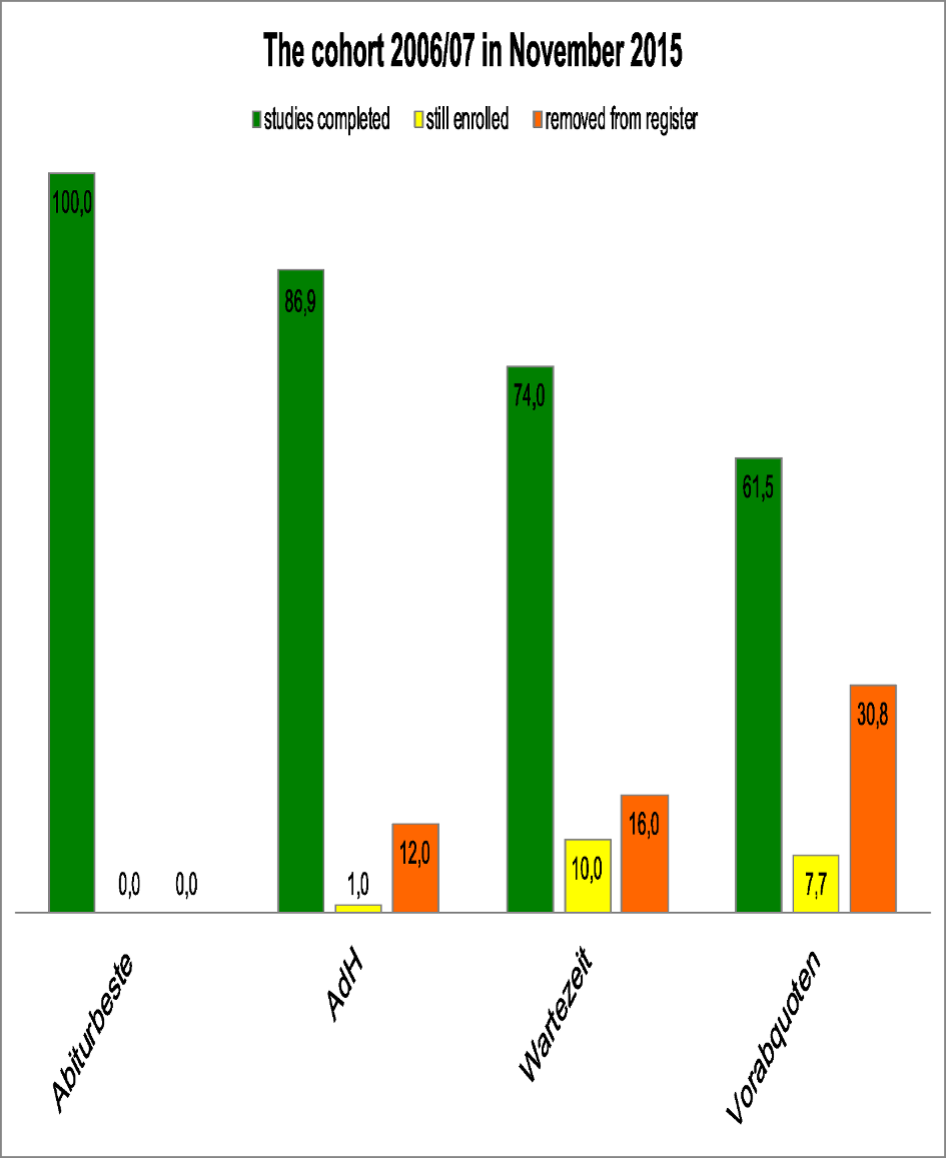
Percentage of students with successful state examination of the study course year 2006/07 by admission groups (status at Nov 2015).

Whether a student has graduated successfully can only be determined after they have passed the third section of the medical examination. This examination is taken after the practical year. Until 2014, students had to take the written M2 examination after the third study section. Now this examination is again set before the practical year. All students of the “Abiturbeste”, who attended university in 2006/07 had, by November 2015, passed all parts of the state examinations. Over 86% of the “AdH” group had definitely successfully completed their studies. Another 12 percent had left the MHH so it was unclear, whether or not they had actually graduated. They either changed universities or broke off their studies. 74% of the “Wartezeit” students had successfully passed their examinations at MHH, whereas 16% had left the university. Even from the group that performed worst in the M1 equivalent examinations, the “Vorabquoten”, 60% had successfully completed their studies three years after the prescribed period of study (see Figure 3 (Fig. 3)).

**Figure 4:**
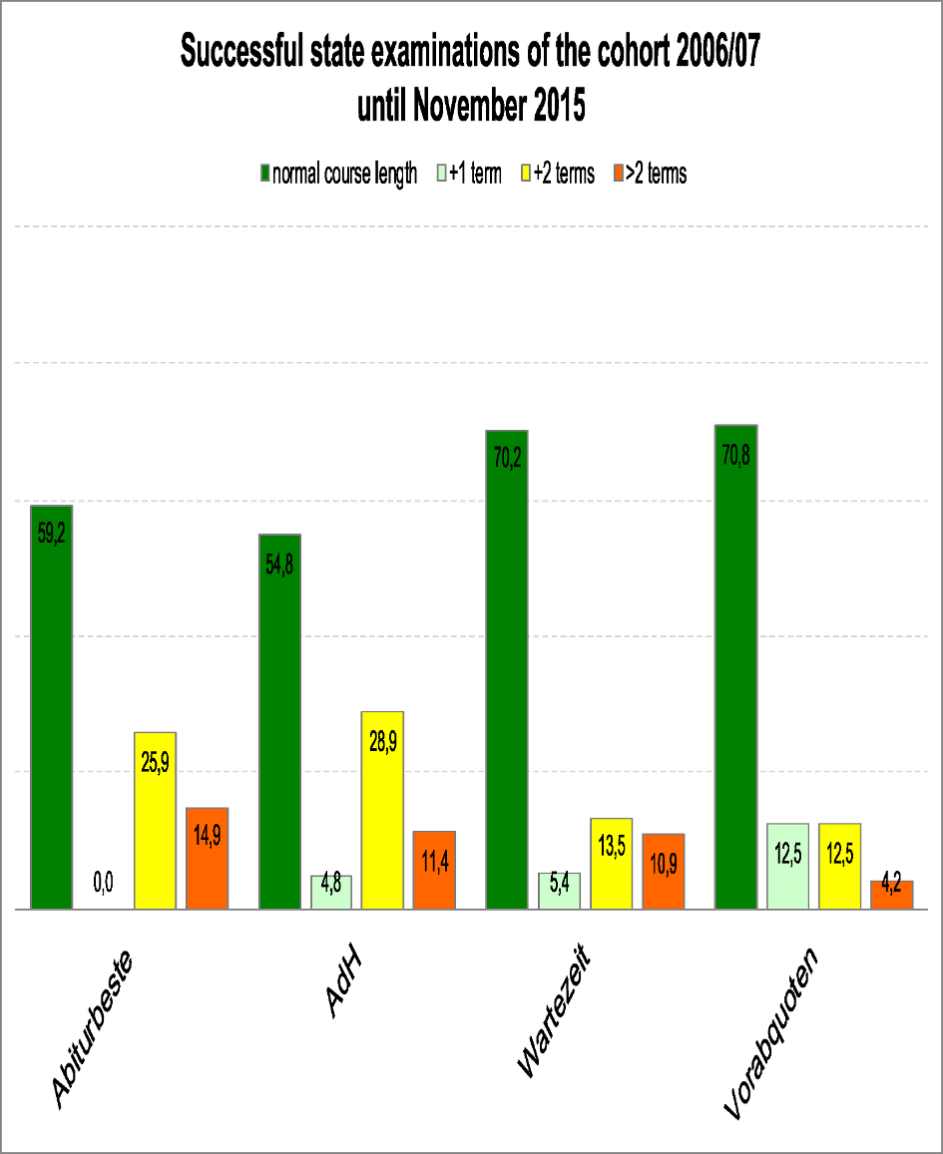
Percentage of students of the study course year 2006/07 finishing successful state examination within the prescribed period of study by admission groups.

Figure 4 (Fig. 4) shows that it was in particular the “Wartezeit” and “Vorabquoten” students who successfully passed their state examination within the prescribed period of study. This is only possible since the M1 equivalence examination is not mandatory for the study progress. On the other hand, the “Abiturbeste” and “AdH” students studied significantly longer, often two or more semesters longer than the minimum necessary. 

**Figure 5:**
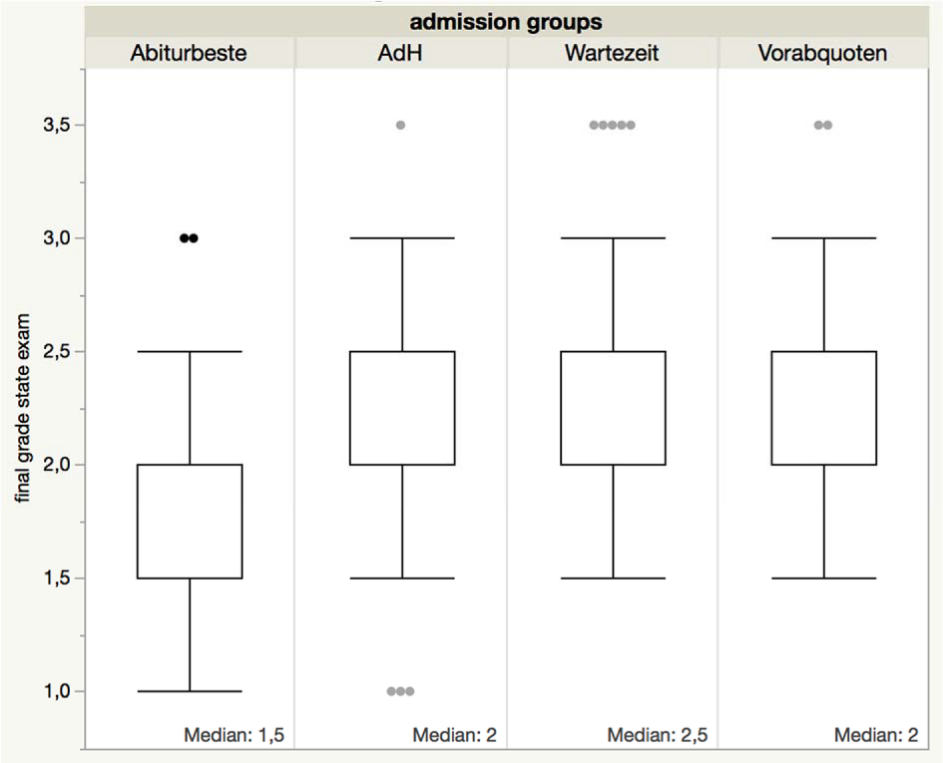
Distribution of state examination grades from November 2015 by admission groups.

When the final grades for the state examination are considered (all admission groups listed separately), there is still a correspondence to the high school examination grade (see Figure 5 (Fig. 5)). Here it should be noted, that the pass rate and grade distribution relate to 27 out of 27 “Abiturbeste” students, 166 out of 191 “AdH” students, 37 out of 50 “Wartezeit” students as well as 24 out of 39 “Vorabquoten” students at the MHH.

## 4. Discussion

According to our data, entering university via “Abiturbeste”, “AdH” procedure or “Wartezeit” and successfully passing the study course correlates at least to the initial qualification. Figure 2 (Fig. 2), figure 3 (Fig. 3) to figure 4 (Fig. 4) show the pass rates and figure 1 (Fig. 1) and figure 5 (Fig. 5) the grade distribution. Heidmann et al. [1] reported that 40% of the “Wartezeit” students did not pass the M1 examination. Our data confirms that this was not due solely to their lesser suitability. The results can be traced back to the missing cut provided by the M1 intermediate examinations. In the model study courses analysed by Heidmann et al. [1] the M1 examination had to be passed before the continuation of studies. Otherwise, the first study block would have to be prolonged for at least another half a year. MHH students, however, lacking one or more equivalent examinations may continue studying. The “Abiturbesten” quota at MHH show better results/score better than the same group of faculties analysed by Heidmann et al [1]. 

The “AdH” quota and the “Vorab” quota students show similar pass rates. Our data contradicts the postulate that the drastic reduction of the “Wartezeit” quota will produce more students graduating within the prescribed period of study. At least at MHH, the “Wartezeit” students complete their studies just as often within the prescribed period of study, as did the students of the “Abiturbeste” (see Figure 4 (Fig. 4)). Their lower grades (see Figure 5 (Fig. 5)), should not count for professional healthcare of the population and for career entry.

The data available does not reveal why “Abiturbeste” and “AdH” students graduate more often at the MHH, but at the same time often study longer than the prescribed period of study – compared to students of “Vorab” or “Wartezeit” quota.

Possible explanations are that they are younger, may start a doctoral thesis and/or take part in a study exchange abroad. It could also relate to less life experience, poorer time management or higher pressure from their surroundings. The independent variables are complex and numerous, as mentioned by Kadmon, Resch, Duelli und Kadmon [9]. 

As Hampe et al. [10] have shown, that selection processes emphasizing specific cognitive performance parameters can reduce the dropout rate in the preclinical study block. This raises the question whether that also applies to motivational parameters. When focusing on the student selection alone it might be forgotten that the dropout rate could be determined by a restrictive idea of academic progress, which causes the prior elimination of certain performance deficits in scientific disciplines to seem like a condito sine qua non for a clinical/ practical education. However in our opinion, you can neither conclude from successful passing of the preclinical study block nor from the kind of university admission whether a doctor will be suited to daily medical practice. The situation is different in regard to the final grade for random samples [4].

In contrast to other current publications [1], [7] we believe it important not to measure academic success through one important intermediate examination or university internal examination, but rather to use the successful passing of the study course as the target criterion. 

Such an approach relativizes the significance of cognitive performance parameters [11]. This confirms the postulation of Conrad, Addams and Young [5] that performance criteria should not be the sole focus in student selection. However, they do not take all factors into account, which influence study progress.

It is apparent that current studies focus very much on the preclinical study block, whereas older surveys also look at the effect of various selection procedures with regard to successful graduation. That is why those older publications in combination with recent international surveys offer a way out from the feared shortage of doctors in the countryside/in rural areas [2], [3], [4], [12]. 

In contrast to e.g. Kadmon, Resch, Duelli & Kadmon [9], we consider it important to also look for structural reasons for academic success, rather than finding explanations in the students solely. Comparisons between current admission quota groups no longer seem necessary as, based on the current judgment of the Federal Constitutional Court [13] regarding the admission process for medical studies, sooner rather than later they will be replaced by a new selection procedure. However, the search for relevant structural variants for medical studies remains current. And if our data can be replicated, it suggests that a change in the structure of the medical study program would allow more students to graduate within the prescribed period of study. In addition, the focus on successful passing of the preclinical study section fails to consider the professional requirements of trained physicians in society. That should not mean that basic knowledge can be neglected nor clinical decisions positively influenced [14].

To show that the academic success of the various admission groups was not a singular effect of one student cohort, we examined the pass rates of various cohorts at the MHH (MHH being the only medical faculty with this study program structure). We found that 74.2% of the “Wartezeit” students from cohort two (beginning: winter semester 2006/07) to cohort four (beginning: winter semester 2008/09) of the model study course, graduated successfully within the prescribed period of study by autumn 2015. Another 6.5% had passed all minor (university-administered) examinations (so-called “Leistungsscheine”), but had not yet passed all sections of the medical state examination. 88.9% of the “Abiturbeste” had passed all “Leistungscheine” examinations as well as all sections of the medical state examination , another 1.1% had passed all “Leistungsscheine” examinations, but not all parts of the medical state examination. Compared with other study courses it can therefore be observed, that even with this large random sample “Wartezeit” students show an impressive rate of success if the five years of their studies up to the practical year are structured continuously and as a single stage process, rather than being divided by the intermediate examination. This means, that even though the quota of students breaking off their medical studies (1) is significantly lower than those of Bachelor’s and Master’s study courses [15], it seems that this rate is even better for MHH students. 

Since the motivation and the qualifications of the students should not differ notably from those investigated by Heidmann et al., we believe that the different structure of the study course can be the only possible explanation for this.

Systematic and more longitudinal analyzes will have to show, not only how success in individual examinations can be optimized but also how the overall rates of successful graduation can be improved. It is already clear that there will not be any simple answer for how to reconcile the complex relationships between expectations of society, individual choice of study, curriculum requirements and academic success.

## Competing interests

The authors declare that they have no competing interests. 

## Figures and Tables

**Table 1:**
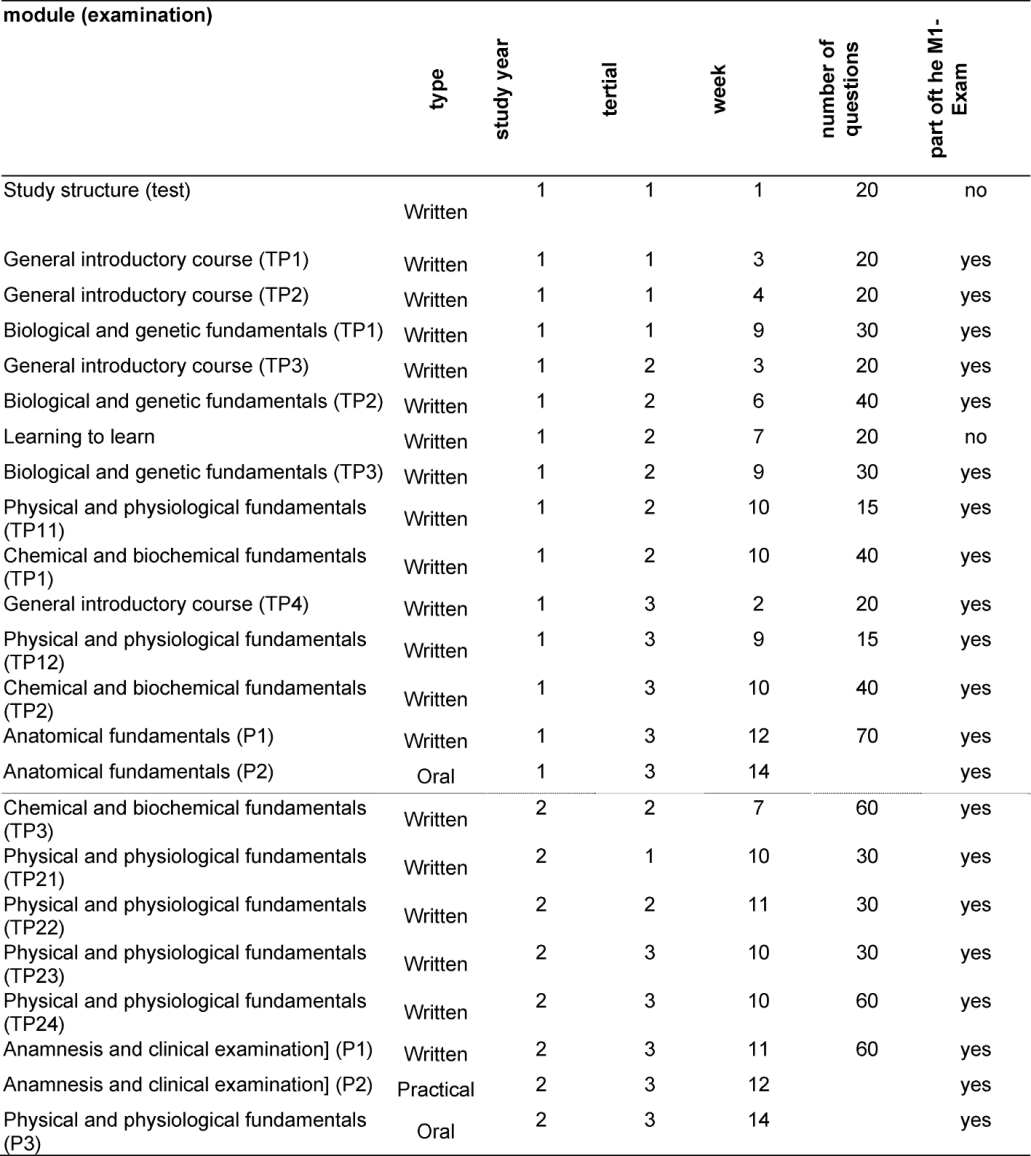
Distribution of examinations of study course years 1 and 2 (2006/07).
